# Supplementary material for: Exploiting the kinesin-1 molecular motor to generate a virus membrane penetration site
Source: Nat Commun. 2017 May 24;8:15496. doi: 10.1038/ncomms15496 (PMC5458101; doi:10.1038/ncomms15496)
Supplement: Supplementary Information — Supplementary Figures and Supplementary Table [file ncomms15496-s1.pdf]

SUPPLEMENTARY INFORMATION

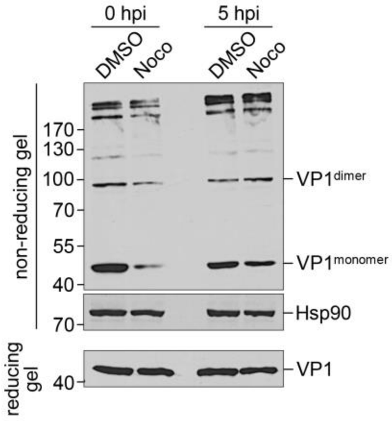

Supplementary Figure 1. CV-1 cells were infected with SV40 (MOI 5) and at 0 hpi or 5 hpi, cells were treated with 1 µM of nocodazole. 12 hpi, cells were lysed, and the resulting whole cell lysate analyzed by non-reducing or reducing SDS-PAGE followed by immunoblotting with the indicated antibodies. The intensity of $VP1^{monomer}$ and $VP1^{dimer}$ in non-reducing SDS-PAGE reflects the amount of virus that has reached ER from the cell surface. This is because SV40's disulfide bonds are reduced when the virus reaches the ER from the cell surface, leading to formation of $VP1^{monomer}$ and $VP1^{dimer}$.

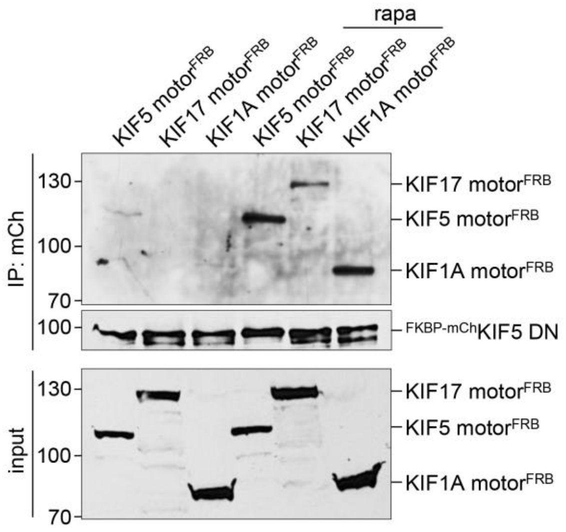

Supplementary Figure 2. COS-7 cells expressing the indicated constructs were treated with or without the rapa linker. After 24 h, cells were lysed and $^{FKBP\text{-}mCh}$KIF5 DN was immunoprecipitated using a mCherry antibody. The precipitated materials were analyzed by immunoblotting using a FRB antibody.

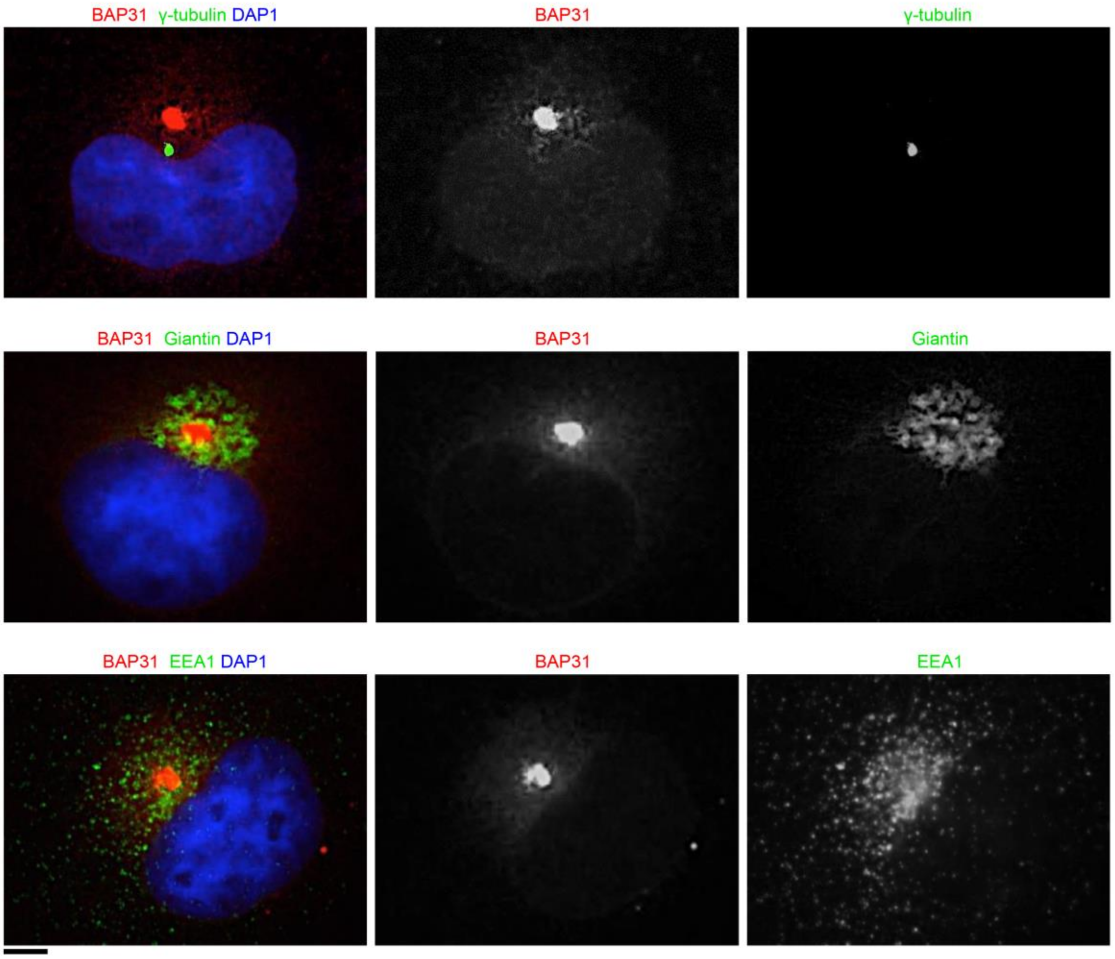

Supplementary Figure 3. CV-1 cells were infected with SV40 (MOI 20). 16 hpi, cells were fixed and stained with the indicated antibodies. Samples were analyzed by immunofluorescence microscopy. Scale bar, 5 µm.

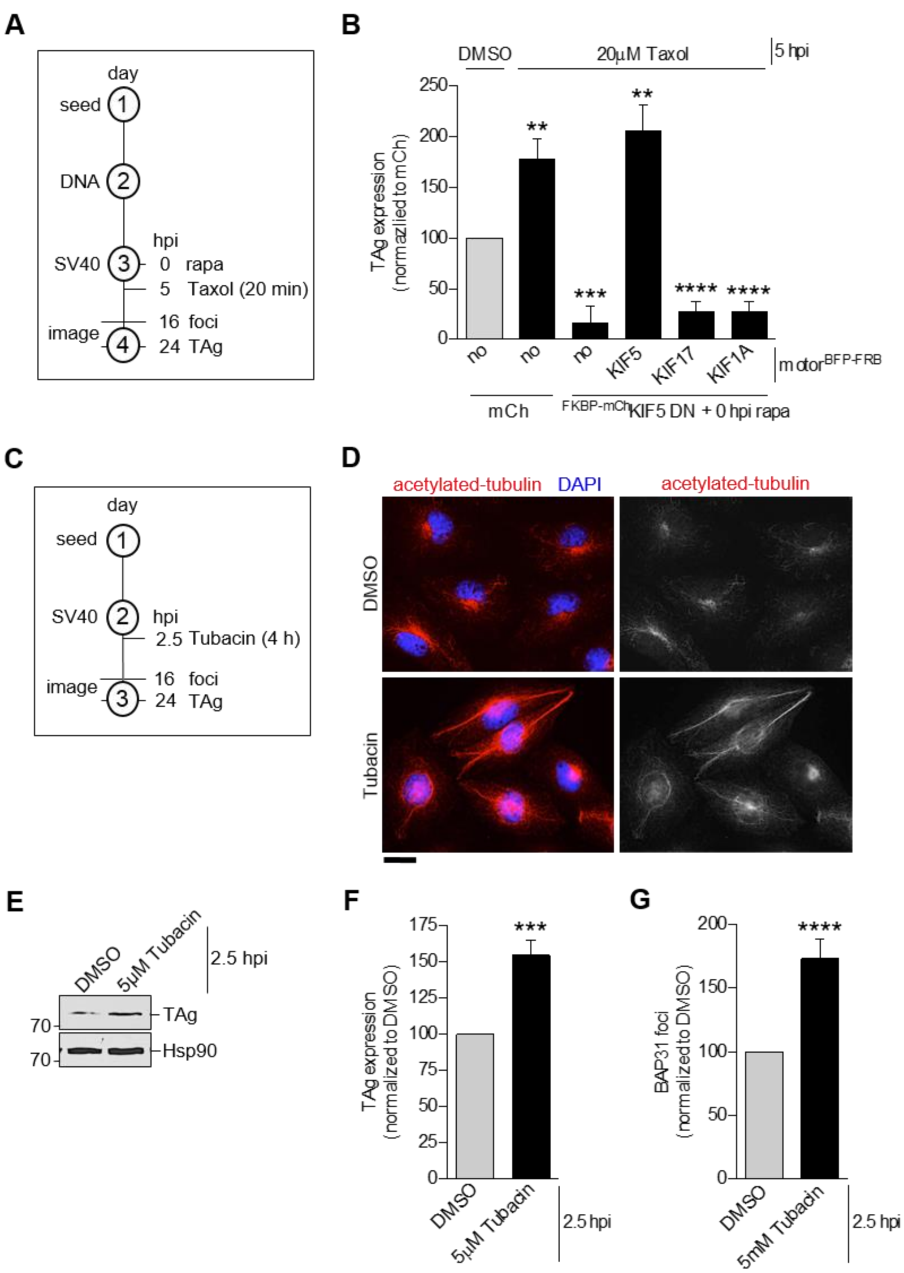

Supplementary Figure 4. A. Diagram depicting the experimental set-up used in panel (B). **B.** CV-1 cells expressing the indicated constructs were infected with SV40 (MOI 0.5). Where indicated, cells were treated with the rapa linker at 0 hpi. 5 hpi, cells were treated with taxol or DMSO control for 20 min and washed. Cells were then fixed at 24 hpi and the percentage of TAg-positive cells was counted only in cells expressing mCh-tagged proteins by immunofluorescence microscopy. Data are normalized to the mCh control (grey bar). Values represent average of the mean (n=3) ± s.d. *P*-values were calculated with the two-

tailed Student's *t*-test; ** $p<0.01$; *** $p<0.001$; **** $p<0.0001$. **C.** Diagram depicting the experimental set-up used in panels (D-G). **D.** Representative images of cells treated with 5 μM tubacin or DMSO for 4 h, and immunostained for acetylated-tubulin. Scale bar, 20 μm. **E.** As in (D), except SV40-infected cells were treated with tubacin and at 48 hpi, the whole cell lysate was immunoblotted with the indicated antibodies. **F.** As in (E), except 24 hpi, cells were analyzed for TAg positive by immunofluorescence microscopy and normalized to DMSO control (grey bar). Values represent average of the mean (n=3) ± s.d. *P*-values were calculated with the two-tailed Student's *t*-test; *** $p<0.001$. **G.** As in (E), except 16 hpi, cells were quantified for the presence of BAP31-positive foci. Values represent average of the mean (n≥3) ± s.d. *P*-values were calculated with the two-tailed Student's *t*-test; **** $p<0.0001$.

Supplementary Figure 5. Uncropped western blots

Figure 1G

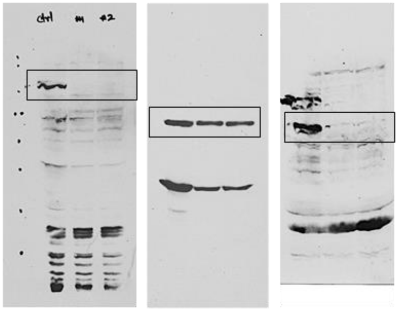

Figure 2A

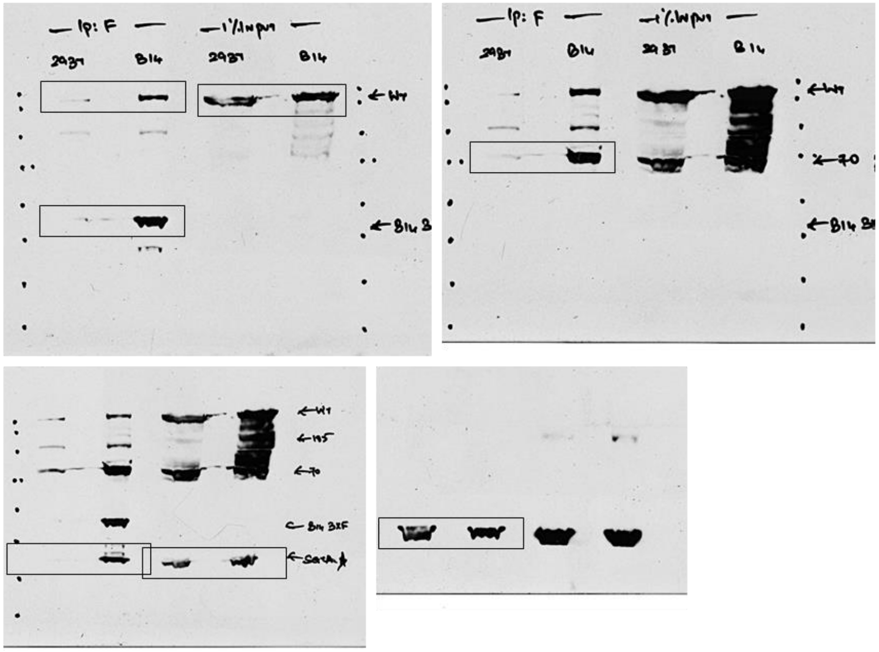

Figure 2B

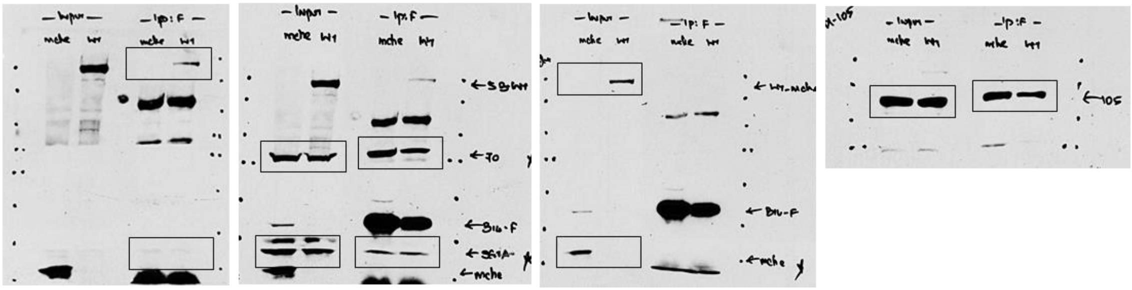

Figure 2C

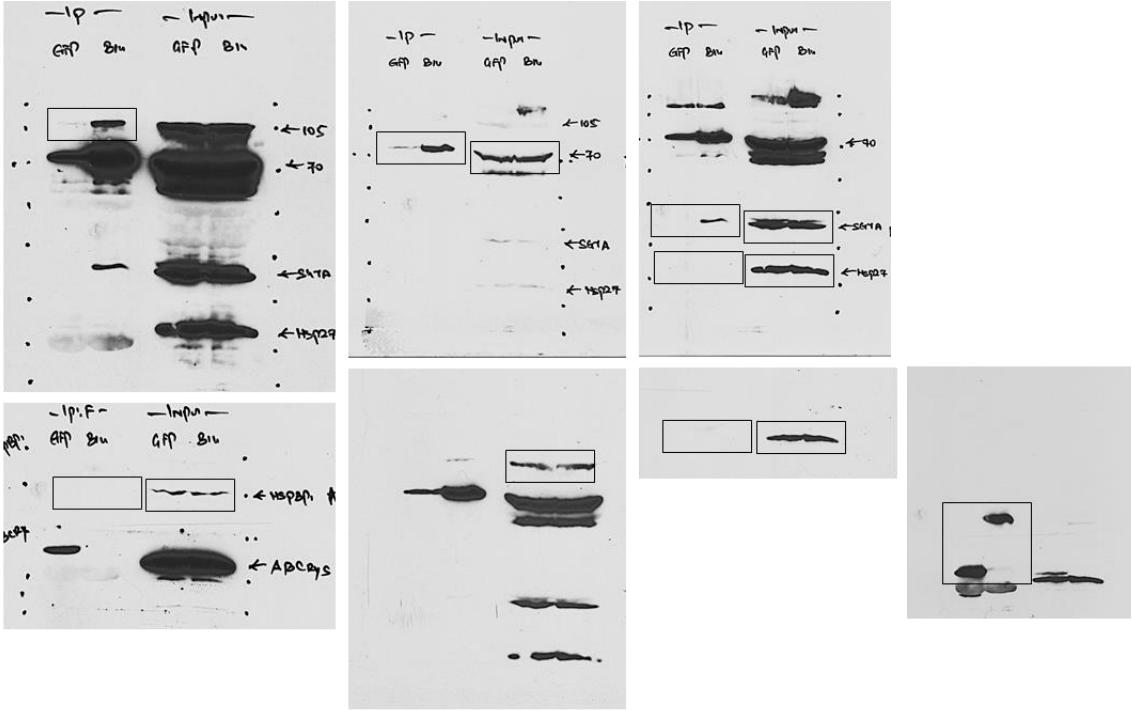

Figure 2D

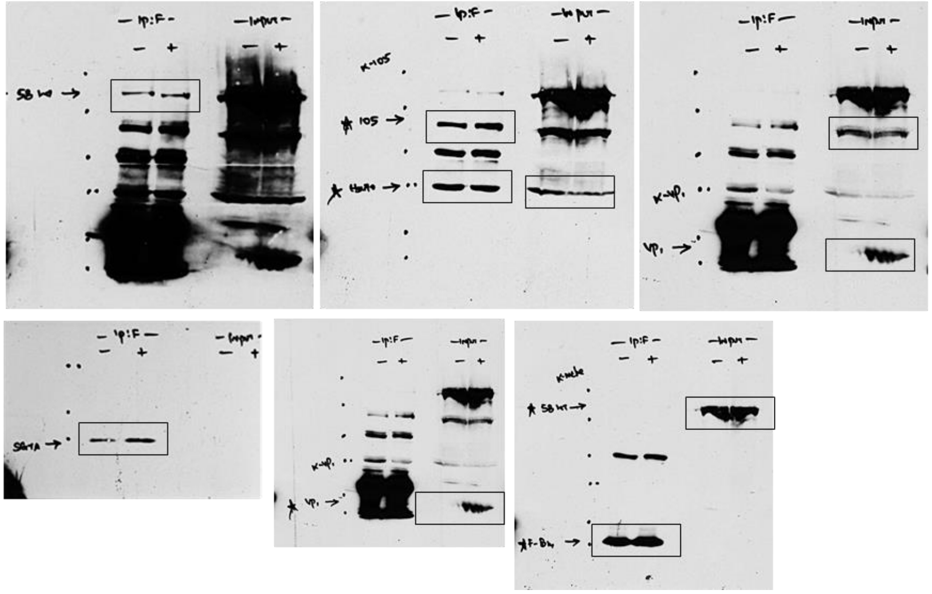

Figure 2E

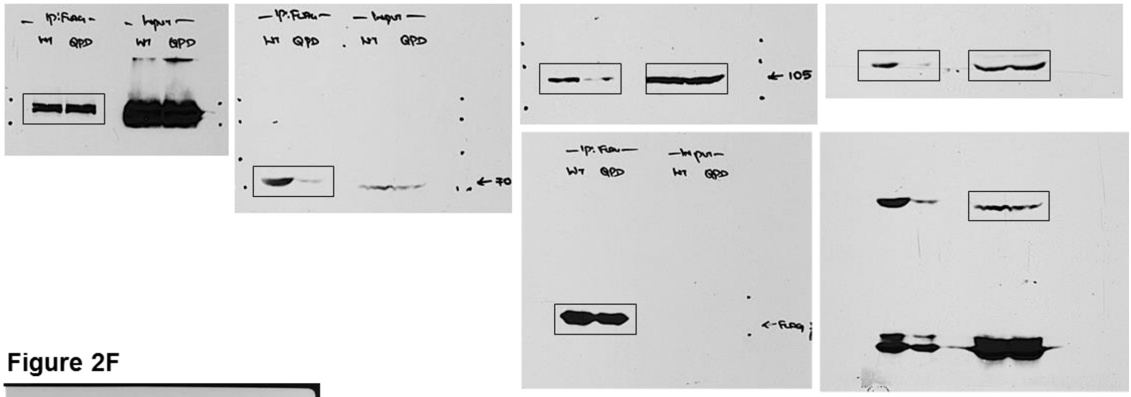

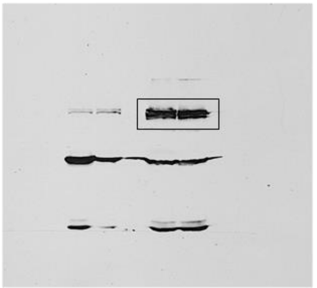

Figure 2F

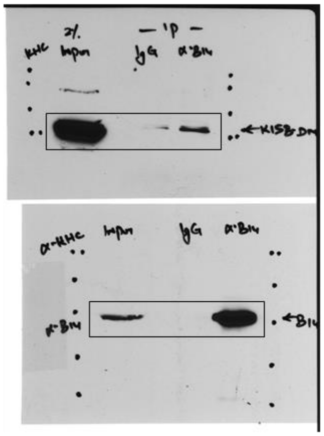

Figure 3A

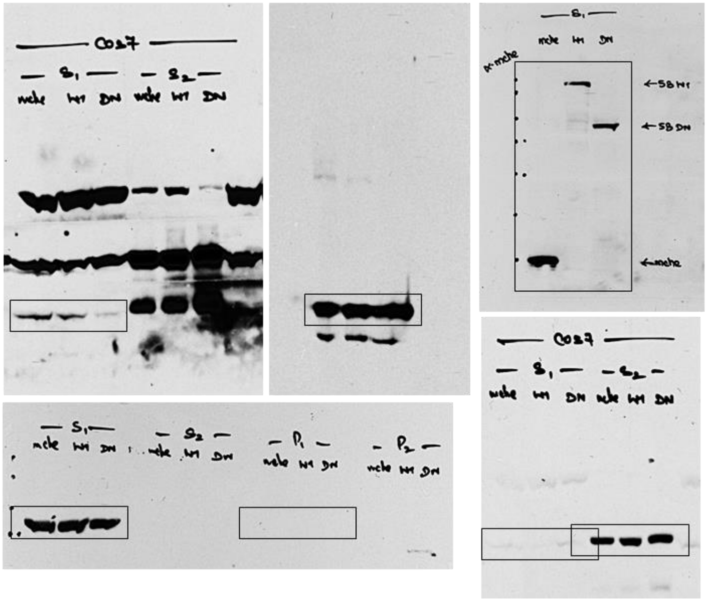

Figure 3B

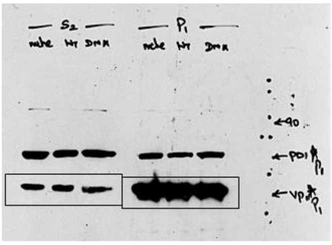

Supplementary Table 1. Antibodies used in this study

antibody	dilution	use	product details
Mouse monoclonal anti-SV40 large TAg	1:500, 1:100	IB, IF	Santa Cruz sc147
Rabbit polyclonal anti-Hsp90	1:3000	IB	Santa Cruz sc7947
Rabbit polyclonal anti-Hsc70	1:2500	IB	Pierce PA5-27337
Rat monoclonal anti-mCherry	1:3000, 1:100	IB, IP	Life Technologies M11217
Mouse monoclonal anti-KIF5 (H2)	1:2500	IB	Millipore MAB1614
Rabbit polyclonal anti-SGTA	1:3000	IB	Proteintech PTG11019-2-AP
Rabbit polyclonal anti-FLAG	1:5000	IB	Sigma F7425
Rabbit polyclonal anti-Hsp105	1:2500	IB	Santa Cruz sc6241
Rabbit polyclonal anti-HspBP1	1:500	IB	Abgent AP14772A
Rabbit polyclonal Hsp27	1:5000	IB	Stressgen SPA-803
Rabbit polyclonal HspB5	1:2000	IB	Stressgen SPA-223
Mouse monoclonal anti-VP1	1:2000, 1:500	IB, IF	Walter Scott (Univ. of Miami)
Rabbit polyclonal anti-B14	1:3000, 1:200	IB, IP	Proteintech PTG11019-2-AP
Mouse monoclonal anti-PDI	1:10000	IB	Abcam ab2792
Rabbit polyclonal anti-CTA	1:3000	IB	Generated in-house
Rat monoclonal anti-BAP31	1:500	IF	Pierce MA3-002
Rabbit polyclonal anti-VP2/3	1:500	IF	Abcam ab53983
Mouse monoclonal anti-tubulin (E7)	1:500	IF	Hybridoma bank (Univ. of Iowa)
Mouse monoclonal anti-acetylated tubulin	1:5000	IF	Sigma T6793
Rabbit polyclonal anti-FRB	1:5000	IB	Enzo Lifesciences ALX215065
Rabbit polyclonal anti-γ-tubulin	1:500	IF	Sigma T3559
Rabbit polyclonal anti-Giantin	1:500	IF	Covance PRB 114c
Mouse monoclonal anti-EEA1	1:500	IF	BD Biosciences 610457
Goat anti-rabbit (HRP conjugated)	1:3000	IB	Sigma A4914
Goat anti-mouse (HRP conjugated)	1:3000	IB	Sigma A4416
Goat anti-rat (HRP conjugated)	1:3000	IB	Sigma A5795
Clean blot IP-detection (HRP conjugated)	1:1000	IB	Thermo 21230
Goat polyclonal anti-rabbit (Alexa flour 488)	1:1000	IF	Life Technologies A11008
Donkey polyclonal anti-rat (Alexa flour 488)	1:500	IF	Life Technologies A11006
Donkey polyclonal anti-rat (Alexa flour 594)	1:500	IF	Life Technologies A21209
Goat polyclonal anti-mouse (Alexa flour 350)	1:100	IF	Life Technologies A11045
Goat polyclonal anti-mouse (Alexa flour 488)	1:1000	IF	Life Technologies A11029
Goat polyclonal anti-mouse (Alexa flour 594)	1:1000	IF	Life Technologies A11032
Rabbit IgG	1:200	IP	Santa Cruz Sc-2027
